# Glycolysis-Driven Prognostic Model for Acute Myeloid Leukemia: Insights into the Immune Landscape and Drug Sensitivity

**DOI:** 10.3390/biomedicines13040834

**Published:** 2025-03-31

**Authors:** Rongsheng Zhang, Wen Jin, Kankan Wang

**Affiliations:** 1 Shanghai Institute of Hematology, State Key Laboratory of Medical Genomics, National Research Center for Translational Medicine at Shanghai, Ruijin Hospital, Shanghai Jiao Tong University School of Medicine, 197 Ruijin Er Rd., Shanghai 200025, China; zhangrongsheng523@163.com (R.Z.); jw11862@rjh.com.cn (W.J.); 2 School of Life Sciences and Biotechnology, Shanghai Jiao Tong University, 800 Dong Chuan Road, Shanghai 200240, China; 3 Sino-French Research Center for Life Sciences and Genomics, Ruijin Hospital, Shanghai Jiao Tong University School of Medicine, 197 Ruijin Er Rd., Shanghai 200025, China

**Keywords:** acute myeloid leukemia, glycolysis, prognostic model, drug sensitivity

## Abstract

**Background**: Acute myeloid leukemia (AML), a malignant blood disease, is caused by the excessive growth of undifferentiated myeloid cells, which disrupt normal hematopoiesis and may invade several organs. Given the high heterogeneity in prognosis, identifying stable prognostic biomarkers is crucial for improved risk stratification and personalized treatment strategies. Although glycolysis has been extensively studied in cancer, its prognostic significance in AML remains unclear. **Methods**: Glycolysis-related prognostic genes were identified by differential expression profiles. We modeled prognostic risk by least absolute shrinkage and selection operator (LASSO) regression and validated it by Kaplan–Meier (KM) survival analysis, receiver operating characteristic (ROC) curves, and independent datasets (BeatAML2.0, GSE37642, GSE71014). Mechanisms were further explored through immune microenvironment analysis and drug sensitivity scores. **Results**: Differential expression and survival correlation analysis across the genes associated with glycolysis revealed multiple glycolytic genes associated with the outcomes of AML. We constructed a seven-gene prognostic model (*G6PD*, *TFF3*, *GALM*, *SOD1*, *NT5E*, *CTH*, *FUT8*). Kaplan–Meier analysis demonstrated significantly reduced survival in high-risk patients (hazard ratio (HR) = 3.4, *p* < 0.01). The model predicted the 1-, 3-, and 5-year survival outcomes, achieving area under the curve (AUC) values greater than 0.8. Immune profiling indicated distinct cellular compositions between risk groups: high-risk patients exhibited elevated monocytes and neutrophils but reduced Th1 cell infiltration. Drug sensitivity analysis showed that high-risk patients exhibited resistance to crizotinib and lapatinib but were more sensitive to motesanib. **Conclusions**: We established a novel glycolysis-related gene signature for AML prognosis, enabling effective risk classification. Combined with immune microenvironment analysis and drug sensitivity analysis, we screened metabolic characteristics and identified an immune signature to provide deeper insight into AML. Our findings may assist in identifying new therapeutic targets and more effective personalized treatment regimes.

## 1. Introduction

Acute myeloid leukemia (AML) is a hematopoietic malignant neoplasm that features the proliferation of genetic mutations and abnormal clones of myeloid progenitor cells. It varies considerably from patient to patient and is associated with poor prognosis and high mortality [1]. Although the treatment of AML has improved over the past decade, problems such as drug resistance and relapse persist [2,3,4]. The assessment of genetic and cytogenetic traits, such as the European Leukemia Network (ELN) categorization system, is currently a major component of AML treatment [5,6,7]. While these approaches can help us understand prognosis to some extent, they have limited utility in guiding personalized treatment, especially in patients without detectable genetic changes. Therefore, we need to find new biomarkers to help us predict the disease more accurately, improve risk stratification, guide treatment choices, and improve survival in patients with AML.

Glycolysis, an important metabolic process for cellular energy acquisition, significantly influences cancer cells and is essential in carcinogenesis and treatment resistance. Cancer cells typically increase their glucose uptake and glycolytic activity to satisfy the energy and metabolic requirements of rapid proliferation, even in a low-oxygen tumor microenvironment. This phenomenon has been termed the “Warburg effect” and has been documented in various cancer types, including AML [8,9,10]. Aberrant glycolysis allows cancer cells to escape drug-induced cell death, which is the underlying cause of treatment failure [11]. Inhibiting glycolytic enzymes like hexokinase 2, lactate dehydrogenase A, and PFKFB3 can slow tumor growth and improve the effectiveness of chemotherapy and radiotherapy [11,12,13,14]. These findings emphasize the essential function of glycolysis in cancer, serving both as a pivotal metabolic process and as a contributor to treatment resistance. This suggests that glycolysis-related molecular markers could serve as valuable prognostic biomarkers.

In recent years, glycolysis has received more attention in AML research. AML cells depend on glucose, so glycolysis is important for their metabolism. Imaging studies show that glucose uptake in bone marrow is much higher in patients with AML using the 18FDG technique [15]. Herst et al. found that elevated aerobic enzyme activity levels at diagnosis are linked to a better treatment response and survival in patients with AML [16]. A correlation between glucose metabolism patterns and reduced survival in patients with AML was identified by Chen et al. [17]. More glycolysis helps AML cells obtain more energy and make intermediates. This enhances their resistance to drug-induced cytotoxicity. It also promotes AML progression by remodeling the tumor microenvironment and enabling immune evasion [17,18,19,20,21]. Patients at high risk of AML often show abnormal glycolysis and a weak immune system. This suggests that glycolysis-related genes (GRGs) may be important markers for predicting AML prognosis [22,23,24,25]. So, glycolysis is a central way AML cells obtain energy. It also plays a crucial role in the initiation, advancement, and therapeutic efficacy of AML. These findings highlight glycolysis as a therapeutic and prognostic target. Even though many investigations have shown the significance of glycolysis in AML and its link to survival [26,27,28,29], the role of glycolysis-driven genetic determinants in AML prognostication remains inadequately explored.

This work employs bioinformatics to explore the mechanistic contribution of glycolysis to AML development and treatment, particularly with a special focus on its prognostic significance. We found important biomarkers by analyzing gene data and built models to predict prognosis. To make predictions more accurate, we combined these models with the ELN classification. We also looked at how immune system interactions affect disease and treatment. At the same time, we tested different drugs to find ones that could improve treatment. Finally, we studied the network of glycolysis-related genes to help create new immunotherapy options. Collectively, this study provides insights into AML pathophysiology and potential biomarkers for improved therapy.

## 2. Materials and Methods

### 2.1. Data Acquisition and Processing

In this study, genes related to glycolysis and their prognostic relevance in AML were analyzed using available public datasets. Glycolysis-driven genetic elements were extracted from the MSigDB compendium [30] and were analyzed for differential expression in the GSE12662 dataset (15 normal and 76 AML samples). Prognosis-related genes were screened against The Cancer Genome Atlas (TCGA)-LAML cohort (n = 150) and used to construct prognostic models. Clinical data, gene expression data, mutation profiles (WES and targeted sequencing), and drug sensitivity data were obtained via the BeatAML2.0 database [31]. Validation of model performance was also performed with two independent RNA sequencing datasets: GSE71014 (n = 104) and GSE37642 (n = 136). Data were analyzed before we removed as many low-quality samples as possible based on predefined quality control criteria (including the proportion of missing values, the presence of outliers, and the completeness of clinical information).

### 2.2. Analysis of Differential Expression and Prognosis

We undertook a quantitative differential expression evaluation employing the limma framework to elucidate genes exhibiting significant and robust expression variations. The inferences extracted from the differential expression analysis were conveyed visually through volcano plots (with thresholds of log_2_FC > 1 and *p* < 0.05) and heatmaps, which were generated by R software (version 4.2.1). The analysis and visualization utilized the following R packages: geoquery (version 2.64.2), limma (version 3.52.2), ggplot2 (version 3.3.6), and ComplexHeatmap. In addition, the identification of prognostic genes was accomplished by integrating TCGA clinical data with the gene expression profiles, followed by Cox regression analysis. Survival regression modeling was performed in batches with the implementation of the survival module in the R program (version 4.2.1) [32].

### 2.3. Construction of a Novel AML Prognostic Risk Model Based on GRGs

We performed univariate Cox regression on prognosis-related glycolysis genes in the TCGA-LAML cohort. A total of 16 prognosis-associated genes (*p* < 0.05) were identified. We used a 10-fold cross-validated LASSO regression to screen the glycolysis-related genes most relevant to prognosis. The value of λ for which the error of prediction for all genes was minimized was used to select the final gene set [33]. Patients were stratified into high- and low-risk groups based on the median risk score, with quartile-based stratification as a supplementary analysis. Kaplan–Meier survival analysis and log-rank tests were used to assess survival differences, while time-dependent ROC curves were generated using the timeROC R package (version 0.4) to evaluate predictive performance. External validation was conducted using BeatAML2.0, GSE37642, and GSE71014. Heatmaps were generated with the ComplexHeatmap package to illustrate molecular, cytogenetic, and clinical characteristics across risk groups, and genetic abnormalities with differential incidences were analyzed using forest plots (forestplot package).

### 2.4. Development of a Nomogram and Calibration Curve

Each probability was calculated and plotted as column–line plots using the RMS package in R to predict the life expectancy of individual subjects, and then a calibration curve was created to check how well the model predicts 1-, 5-, and 10-year survival in patients with AML.

### 2.5. Immune Infiltration Analysis

The R software packages CIBERSORT (version 1.03), GSVA (version 1.44.5), and xCell (version 1.1.0) were employed to measure the corresponding proportions of various infiltrating immune cell types in different samples. Together, these methods provided a detailed profile of immune infiltration in AML samples.

### 2.6. Drug Sensitivity Analysis

We assessed drug sensitivity scores for each risk group using the R software packages oncoPredict (version 0.2) and pRRophetic (version 0.4). Scores were based on drug and cell line data from the GDSC database. The half maximal inhibitory concentration (IC50) values for each drug in the two groups were then compared using Wilcoxon tests in R (version 4.3.2). IC50 values are inversely related to drug sensitivity. This helps us to observe how patients with AML within various risk groups are responding to treatment.

### 2.7. Differentially Enriched Genes and PPI Network Interaction

Differential gene analysis was conducted by using gene expression data normalized to TPM. Using a model of seven glycolysis-related genes (7-GRGs), we applied the limma R package. The selected genes required an absolute log_2_FC > 1 and a corrected *p*-value < 0.05. The results were displayed in volcano plots. Using the clusterProfiler program, the functional enrichment study of many differently expressed genes was performed using gene ontology (GO) and the Kyoto Encyclopedia of Genes and Genomes (KEGG) pathways. Furthermore, we conducted gene set enrichment analysis (GSEA) utilizing ClusterProfiler (version 4.4.4) and illustrated the findings through volcano plots. Finally, STRING website (https://string-db.org/) and Cytoscape (version 3.10.3) tools were applied to construct protein–protein interaction (PPI) networks, emphasizing the relationships between key genes and their possible biological relevance.

### 2.8. Statistical Analyses

Statistical assessments were performed using the R computational platform. Differences were evaluated with the Wilcoxon rank-sum test and the Student’s *t*-test. For comparisons of risk scores with various clinical variables (clinical and molecular features, including AML subtypes and the 2017, 2022, and 2024 ELN classifications), we executed a univariate analysis of variance (ANOVA). Survival outcomes between risk groups were assessed using Kaplan–Meier analysis with log-rank testing.

## 3. Results

### 3.1. Screening of Glycolysis-Related Prognostic Genes in AML

**Figure 1:**
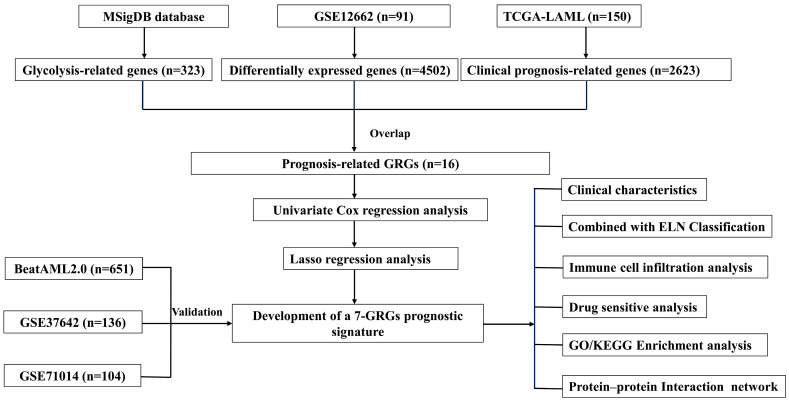
Outline of the analyses performed in this study.

**Figure 2:**
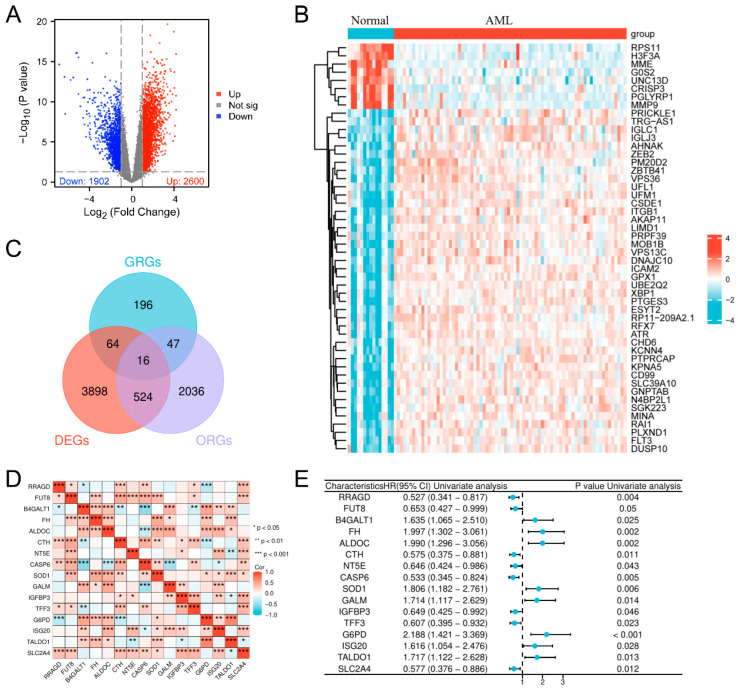
Comprehensive evaluation of variably expressed, glycolysis-associated predictive biomarkers in AML. (**A**) Volcano plot depicting fluctuating gene expression between samples from patients with AML and normal controls, with elevated genes depicted in red and downregulated genes in blue (|log_2_FC| > 1, *p* < 0.05). (**B**) Heatmap illustrating the DEGs between AML and normal samples. (**C**) Venn diagram illustrating the overlap of DEGs, ORGs, and GRGs. (**D**) Correlation heatmap of prognostic metabolic genes pertinent to glycolysis in AML. (**E**) The association between the expression of genes participating in glycolytic flux and prognosis in AML was evaluated using univariate Cox regression.

The comprehensive progression associated with this analysis is illustrated in Figure 1. We implemented a differential gene expression inspection of the GSE12662 dataset (containing 15 normal samples and 76 AML samples) and detected a sum of 4502 genes with differential expression (DEGs), where 2600 were upregulated and 1902 were downregulated, with screening thresholds of |log_2_FC| > 1 and *p* < 0.05 (Figure 2A,B). We identified a total of 323 GRGs from the MSigDB database. Subsequently, Cox regression analysis of the expression profiling of people with AML in the TCGA database identified 2623 genes associated with OS. Upon intersecting the DEGs, GRGs, and OS-related genes (ORGs), 16 glycolysis-related prognostic differentially expressed genes were obtained (Figure 2C). Analysis of the correlations among these 16 genes revealed notable positive and negative relationships, indicating their potential role in jointly regulating the glycolytic pathway in AML (Figure 2D). Univariate Cox regression analysis identified these 16 genes as substantially associated with OS (*p* < 0.05), highlighting their potential prognostic significance in AML (Figure 2E).

### 3.2. Development of a Prognostic Risk Evaluation Model Integrating Glycolysis-Dependent Genes in AML

**Figure 3:**
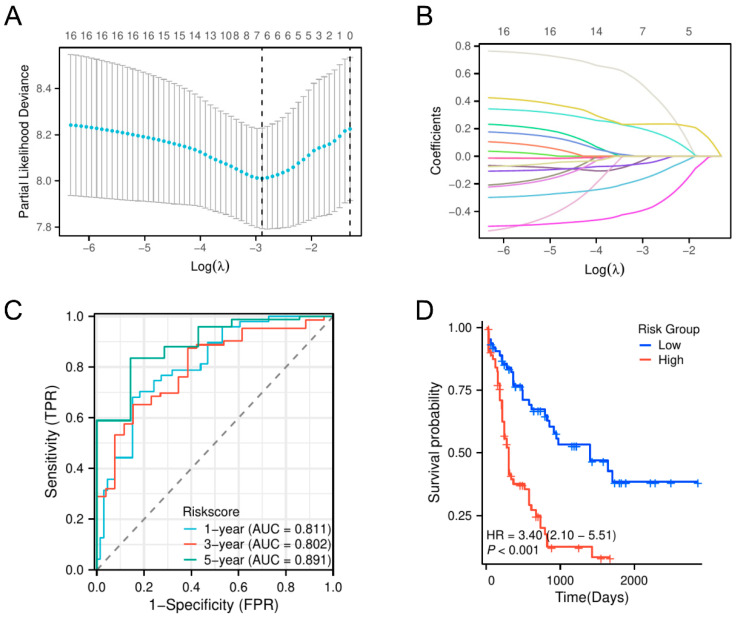
Establishment of a GPM. (**A**) The λ parameter represents the coefficients of the chosen features. The colorful lines indicate the cross-validation error for different λ values, where the dotted line marks the optimal λ. (**B**) Development of a glycolysis-based prognostic model using the LASSO algorithm for patients with AML. The colorful lines represent the trajectories of gene coefficients as the penalty parameter (λ) changes. (**C**) The time-dependent ROC test showed that the AUCs at 1-year, 3-year, and 5-year intervals demonstrated the prognostic model’s ability to predict OS in both high-risk and low-risk patient groups. (**D**) TCGA-cohort KM survival analysis using risk scores.

We then built a strong AML prognostic model by LASSO regression analysis based on these 16 glycolysis-related prognostic genes. This prognostic model consisted of seven prognosis-related genes associated with glycolysis (*G6PD*, *TFF3*, *GALM*, *SOD1*, *NT5E*, *CTH*, and *FUT8*) (Figure 3A). We visualized the association between the model features and the patient’s prognostic outcomes and validated the robustness of the selected variables by trajectory analysis (Figure 3B). A table summarizing known inhibitors for each gene and their potential clinical applications is provided in Supplementary Table S1. Using ROC analysis across time, we evaluated the model’s predictive ability and derived AUC values of 0.811, 0.802, and 0.891 for 1, 3, and 5 years, respectively (Figure 3C). These results validated the model’s discriminative strength and stability in predicting long-term survival outcomes. The Kaplan–Meier survival curves for OS indicated an important distinction between the two risk categories (HR = 3.4, *p* < 0.01), with individuals in the group with the greatest risk exhibiting a poor prognosis (Figure 3D). Additionally, we performed a Kaplan–Meier survival analysis based on quartile stratification of risk scores, which further validated the prognostic value of the model across different risk groups (Supplementary Figure S1). Furthermore, a heatmap was generated to examine the distribution of clinical parameters across risk groups, revealing significant differences in age, white blood cell count, FAB classification, and cytogenetic risk between high- and low-risk groups, suggesting their close association with AML prognosis. Other clinical parameters, such as platelet count, bone marrow and peripheral blood blast cell proportions, and sex, did not show significant differences (Supplementary Figure S2).

**Figure 4:**
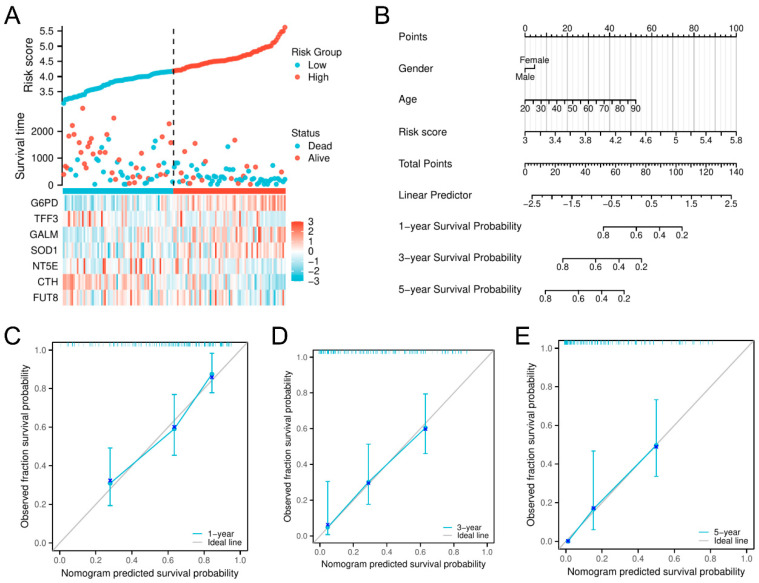
Correlation between clinical attributes and GPM in the model development group. (**A**) The scattering of risk values, the clinical prognosis, and the heatmap of 7-GRGs within the GPM. The dashed line separates the low-risk group from the high-risk group. (**B**) Nomogram integrating risk scores with pathologic features. (**C**–**E**) Calibration curves for predicting 1-, 3-, and 5-year overall survival in patients with AML.

To provide insight into the clinical and biological implications of this model, we developed a risk factor plot to demonstrate how risk scores, survival outcomes, and gene expression profiles differed between risk groups (Figure 4A). *G6PD*, *GALM*, and *SOD1* had higher expression levels in the high-risk cohort, whereas *TFF3*, *NT5E*, *CTH*, and *FUT8* were upregulated in low-risk patients. To evaluate the relative importance of the model’s clinical pertinence, we constructed a nominative representation of the risk level and identified key clinical aspects. This model predicts personalized survival outcomes (Figure 4B). In the nomogram, specific points are assigned based on each feature, and personalized survival probabilities can be calculated for patients with AML. The calibration plots indicated that the map effectively predicted the outcomes at 1, 3, and 5 years in terms of real survival, hence reinforcing the model’s precision and dependability (Figure 4C–E). A glycolysis-related prognostic model (GPM) to partition patients with AML according to survival risk was developed.

### 3.3. Validation and Prognostic Assessment of the Glycolysis-Related Prognostic Model (GPM) for Overall Survival in AML Across Multiple Independent Datasets

**Figure 5:**
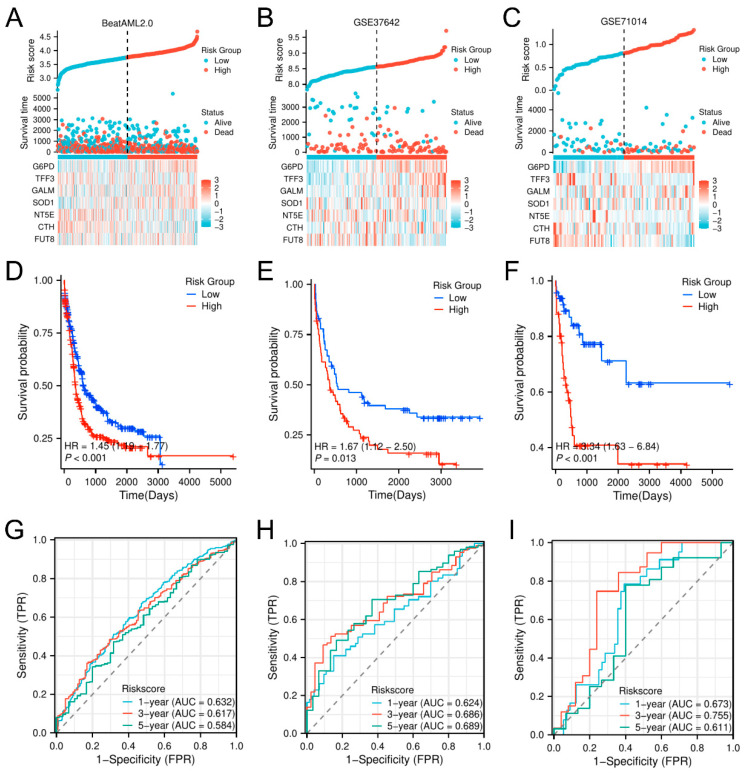
Verification of the GPM model for OS prediction in distinct datasets. (**A**–**C**) Risk factor plots for the BeatAML2.0, GSE37642, and GSE71014 datasets, showing risk scores, OS status, gene expression levels, and cut-off points for classifying high- and low-risk groups. (**D**–**F**) Kaplan–Meier plots were used to compare OS between risk groups across the BeatAML2.0, GSE37642, and GSE71014 datasets. (**G**–**I**) The predictive accuracy of risk scores for OS was assessed by 1-, 3-, and 5-year time-dependent ROC curves in the BeatAML2.0, GSE37642, and GSE71014 datasets.

To evaluate the prognostic significance of the glycolysis-related predictive model (GPM) for AML, we utilized the framework across three separate databases: BeatAML2.0, GSE37642, and GSE71014. We created risk factor plots to illustrate the patterns associated with risk scores, longevity status, and expressed gene levels, and similarly, we stratified patients into elevated and diminished risk cohorts based on the median risk score. Our validation datasets exhibited gene expression patterns highly similar to TCGA, with only minor discrepancies (Figure 5A–C). KM analysis verified that individuals at heightened risk had significantly worse OS in all datasets (BeatAML2.0: HR = 3.4, *p* < 0.01; GSE37642: HR = 3.2, *p* < 0.01; GSE71014: HR = 3.6, *p* < 0.01), reinforcing the robustness of our model (Figure 5D–F). Time-dependent ROC analysis further supported its predictive accuracy, with AUC values of 0.811, 0.802, and 0.891 in BeatAML2.0 and 0.803, 0.790, and 0.875 in GSE71014 (Figure 5G–I). These results emphasize the strong predictive power of the model across multiple independent datasets and its outstanding performance in long-term survival prediction. Taken together, our results suggest that the GPM is an effective tool for predicting OS in patients with AML and performs consistently across cohorts, providing a reliable basis for individualized prognostic assessment and treatment strategies.

### 3.4. Enhancement of AML Prognosis Prediction by Combining the Glycolysis-Related Model with the ELN Classification

**Figure 6:**
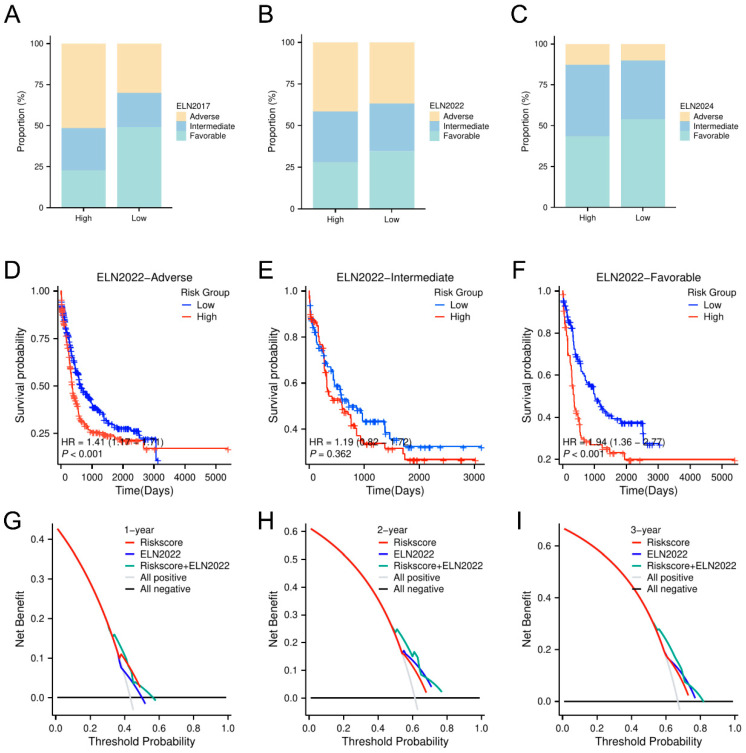
Prognostic stratification of AML based on the ELN classification and risk model. (**A**–**C**) Histograms showing the changes in the ELN 2017, 2022, and 2024 subgroups. (**D**–**F**) KM curves assessing OS within the ELN 2022 subgroups. (**G**–**I**) DCA was performed for AUC estimations at 1-, 2-, and 3-year intervals.

To provide a comprehensive picture of the gene mutation spectrum associated with the model, we examined common driver mutations and chromosomal abnormalities in the BeatAML2.0 training set (Supplementary Figures S3–S5). The mutation frequencies of *DNMT3A*, *ASXL1*, *SF3B1*, and *MLL* translocation were higher in the high-risk group, whereas the frequencies of *CBFB::MYH11*, *PML::RARA*, and *biallelic CEBPA* (*biCEBPA*) mutations were more common in the low-risk group. In addition, we combined the GPM with a practical risk stratification system in AML—the ELN—to evaluate whether it improved risk stratification. Using genetic and cytogenetic factors, the ELN classification breaks patients into various risk categories. As expected, according to the ELN 2017 classification, patients in the high-risk group exhibited a significantly higher rate of adaptation to the adverse subgroup, denoting its ability to robustly differentiate patients at high vs. low risk (Figure 6A). Though the proportion of individuals in the adverse subgroups was less pronounced in the ELN 2022 and ELN 2024 classifications, a consistently inferior prognosis was seen between the high-risk and the low-risk groups, which demonstrated the consistency of our model across different ELN classification platforms (Figure 6B–C). KM analysis indicated that, in both the adverse and favorable ELN 2022 subgroups, the low-risk cohort had conspicuously better survival trajectories than the high-risk cohort, thus further validating the model in discarding higher-risk patients in each of the ELN risk categories (Figure 6D–F). In the intermediate group of ELN 2022, although no significant survival differences were observed, the survival differences seen in other subgroups further corroborated IUH’s highly predictive power. We performed a decision curve analysis (DCA) to evaluate the combined model’s clinical value. Refining the prognostic model by integrating it with the ELN 2022 classification revealed a higher net benefit for several risk thresholds, particularly for a high risk of the targeted dataset, confirming the real clinical utility of the optimized approach to survival prediction (Figure 6G–I).

### 3.5. Immune Microenvironmental Variances Between the High-Risk and Low-Risk Classifications Uncover Immune Downregulation and Anti-Oncogenic Activity

**Figure 7:**
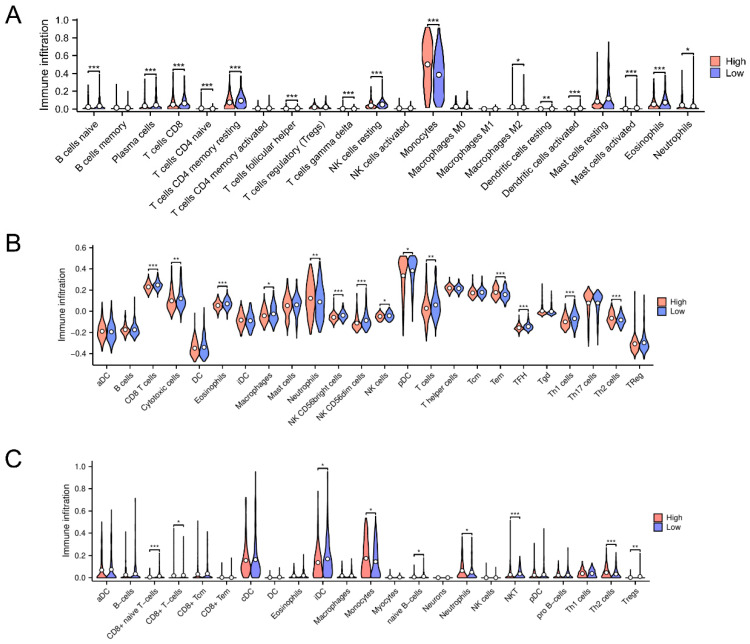
Immune cellular constitution and penetration trends in elevated- and diminished-risk AML cohorts. (**A**–**C**) The differences between immune cells, evaluated by CIBERSORT, ssGSEA, and xCELL. Data were analyzed for statistical significance, where * represents *p* < 0.05, ** represents *p* < 0.01, and *** represents *p* < 0.001.

To determine if our prognostic model could stratify patients and whether clinical outcomes can be linked to immune microenvironment variation, we calculated immune infiltration analyses through CIBERSORT, ssGSEA, and xCell. In the study, monocyte abundance was significantly high, while T cells were substantially distinctly suppressed in the high-risk cluster compared to the low-risk cluster according to the CIBERSORT analysis (Figure 7A). SsGSEA analysis showed that neutrophil infiltration was among the high-risk strata, while T cells and Th1 subsets exhibited pronounced attenuation (Figure 7B). In line with these results, the xCell analysis revealed enhanced monocyte infiltration and a significant decrease in T cells and iDCs, which are crucial to initiating anti-tumor responses (Figure 7C). These findings suggest a potent immunosuppressive microenvironment in the high-risk group, with elevated monocyte and neutrophil infiltration but reduced T cells, Th1 cells, and iDCs. On the contrary, the low-risk group presented a more immunoactive microenvironment, which might account for its superior clinical outcomes. Taken together, these findings indicate that the prognostic model effectively stratifies patients according to survival risk and offers insight into differences in the immune microenvironment, thus establishing a foundation for the development of immunotherapeutic strategies for AML.

### 3.6. Identification of Potential Therapeutic Agents for AML Using Prognostic Model-Based Drug Sensitivity Analysis

**Figure 8:**
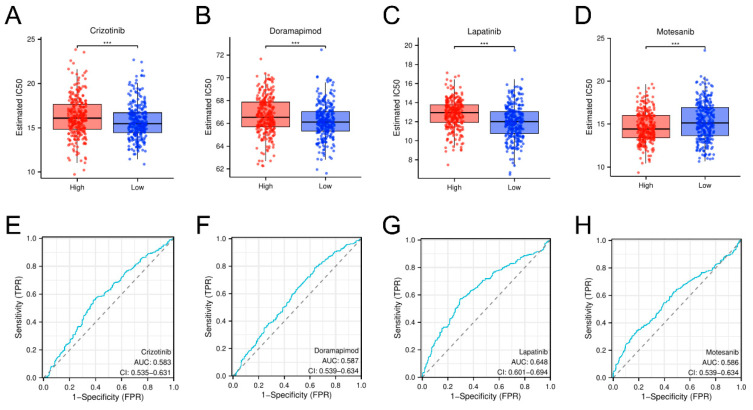
Drug sensitivity analysis. The graphical distributions and ROC evaluations highlight the differences in drug response to crizotinib (**A**,**E**), doramapimod (**B**,**F**), lapatinib (**C**,**G**), and motesanib (**D**,**H**) between the high-risk and low-risk populations identified by the GRG methodology. The blue line represents the ROC curves of the different drug prediction models (AUC values reflecting prediction accuracy), and the gray dashed line (AUC=0.5) is a random reference line indicating a baseline of prediction without discriminatory power. Data were analyzed for statistical significance, where *** represents *p* < 0.001.

Next, we used our prognostic model to uncover potential drugs to address the therapeutic challenges in AML using 166 compounds from the BeatAML2.0 and GDSC databases. High IC50 values for crizotinib, doramapimod, and lapatinib among the high-risk subset indicated drug resistance, and in contrast, low IC50 values for motesanib predicted enhanced sensitivity (Figure 8A–D). ROC curve analysis confirmed these results (AUC of 0.583, 0.587, 0.648, and 0.586 for crizotinib, doramapimod, lapatinib, and motesanib, respectively, Figure 8E–H). Lapatinib showed the highest AUC, indicating the model’s strong predictive power for drug responses. These results underscore the promise of our model to inform personalized AML therapy.

### 3.7. Differential Gene Expression and Functional Enrichment Analyses Reveal IL-6 as a Key Mediator in High-Risk AML

**Figure 9:**
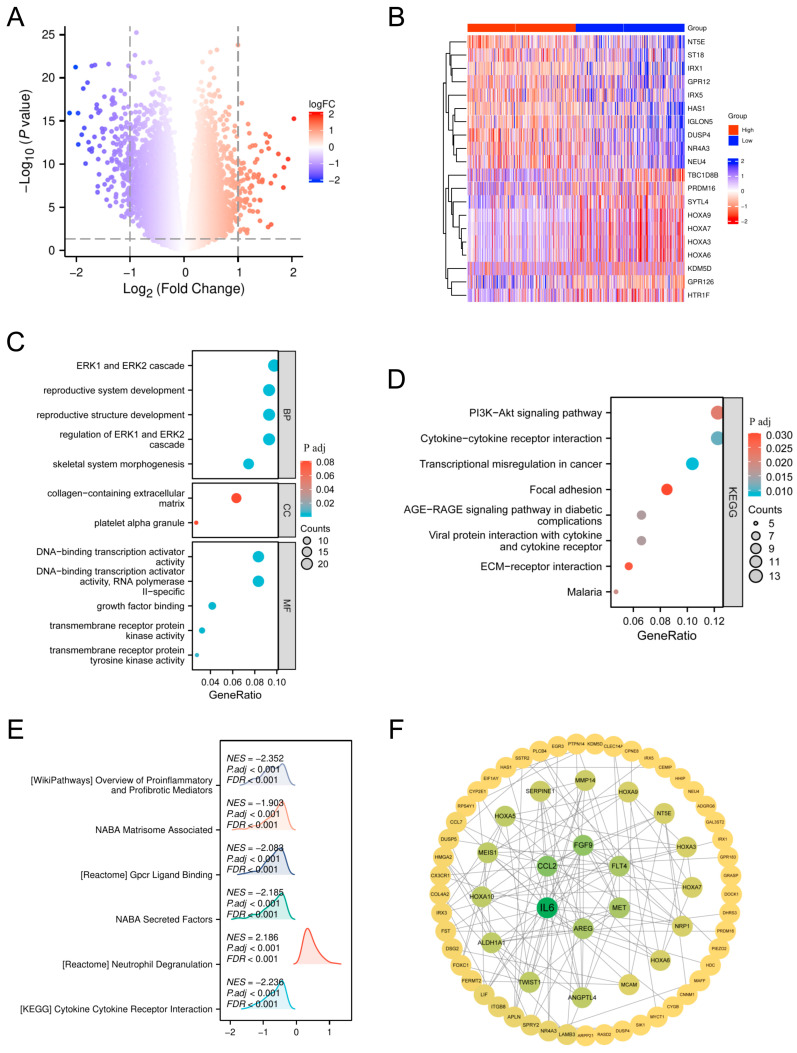
Recognition of central genes and molecular pathways in elevated-risk AML: contrasting expression profiles, biological enrichment, and interactome analysis. (**A**) DEGs between high- and low-risk AML. (**B**) Top 20 DEGs in high- and low-risk AML. (**C**) GO enrichment analysis of DEGs in high-risk AML. (**D**) KEGG pathway enrichment of DEGs in high-risk AML. (**E**) GSEA reveals enriched pathways in high-risk AML. (**F**) PPI network analysis identifies key molecular interactions in the high-risk group.

To further explore the underlying mechanisms between the high- and low-risk groups, we performed differential expression gene (DEG) analysis using a threshold of |log_2_FC| > 1, *p* < 0.05. This study identified 246 DEGs, including 160 upregulated and 86 downregulated genes (Figure 9A). Among the top 20 differently expressed genes, some were linked to important processes connected to diseases (Figure 9B). We carried out GO-based functional enrichment analysis and KEGG pathway mapping to elucidate the biological relevance of these DEGs. The data indicated that the differentially expressed genes were significantly associated with the ERK1/2 signaling pathway and the PI3K-Akt cascade (Figure 9C,D). Furthermore, GSEA-identified pathways were greatly enhanced within the intensified-risk cohort, suggesting molecular causes for poor prognosis (Figure 9E). Finally, a PPI network analysis of DEGs (log_2_FC > 1.2, log_2_FC < −1.2) identified IL-6 as the most central gene in the network, highlighting its potential central role in the molecular mechanisms of the elevated-risk cohort and its contribution to an unfavorable prognosis.

## 4. Discussion

AML is a highly diverse blood cancer distinguished by the abnormal growth and development of myeloid precursor cells within bone marrow [1,34]. The standard treatment in AML remains chemotherapy, comprising agents like cytarabine and daunorubicin or idarubicin [35]. Although these treatments show efficacy in the short term, more than 60% of patients with AML ultimately experience relapse [36]. Metabolic modulation has been shown to augment the susceptibility of neoplastic cells to chemotherapeutic agents, as cancer cells often exhibit an enhanced glycolytic signaling pathway to supply energy [8,37]. Multiple studies have shown that abnormalities in glycolysis are associated with the development of AML [38,39]. In the current investigation, we introduce a prognostic framework based on glycolysis-associated genetic markers to effectively distinguish patients at higher and lower risk of AML and validate its survival prediction in multiple datasets. The model combined with the ELN classification improves the accuracy of risk stratification, and the immune microenvironment analysis reveals the immunosuppressive characteristics of the high-risk group. Drug sensitivity profiling identified candidate therapies for personalized treatment, further supporting the potential of the model for clinical application.

The prognostic model we developed is primarily based on seven genes involved in the glycolytic signaling pathway: *G6PD*, *TFF3*, *GALM*, *SOD1*, *NT5E*, *CTH*, and *FUT8*. *G6PD* encodes glucose-6-phosphate dehydrogenase, which, as a catalytic enzyme in the pentose phosphate pathway, plays a key role in maintaining cellular redox homeostasis and promoting AML cell proliferation [40]. The targeted inhibition of G6PD increases tumor sensitivity to chemotherapy, particularly when the NRF2 pathway is activated, offering a potential strategy for anticancer therapy [41,42,43,44,45]. Studies have shown that *G6PD* mutations in patients with AML may lead to redox imbalance, which, in turn, affects tumor cell growth and drug resistance, especially in FLT3 inhibitor therapy, where G6PD-driven redox metabolism promotes the development of drug resistance [40,46]. In addition, *G6PD* deficiency has been associated with an increased risk of invasive fungal infections, further suggesting its potential importance in the prognostic assessment of patients with AML [46]. The protein encoded by *TFF3* is a growth-promoting mucin involved in regulating the progression of several cancers, including gastric, breast, and colorectal cancers. It promotes cancer cell proliferation, metastasis, and chemotherapy resistance by interacting with the CD147 receptor and modulating signaling pathways, and it has predictive value in the endocrine treatment response in breast cancer [46,47,48,49]. The *GALM*-encoded glucose-1-phosphate translocase plays a key role in glucose metabolism and drives glioma cell proliferation and tumor progression by promoting alterations in glucose metabolic pathways [50]. Superoxide dismutase encoded by *SOD1* is an important antioxidant enzyme that scavenges intracellular reactive oxygen species (ROS) and maintains cellular redox balance. It promotes tumor cell proliferation, survival, and metastasis by regulating ROS levels in cancer, and as a novel target, the inhibition of SOD1 expression may be effective in slowing down cancer progression [51,52,53,54]. The *NT5E*-encoded CD73 protein is an ecto-5′-nucleotidase that converts adenosine triphosphate (ATP) to immunosuppressive adenosine, thereby modulating the immune response. In acute myeloid leukemia (AML), CD73 has emerged as a potential therapeutic target by promoting immune escape in the tumor microenvironment and driving AML cell proliferation and metastasis [55,56]. The CTH-encoded protein cysteine γ-cleaving enzyme plays a key role in thioamino acid metabolism and is involved in the generation of hydrogen sulfide and the regulation of intracellular antioxidant responses. In cancer, CTH is an important regulator of tumor progression by modulating the redox balance of tumor cells and promoting proliferation and metastasis, and its inhibition significantly suppresses the formation of several cancers such as glioblastoma [57,58,59,60]. The α-(1,6)-fucosyltransferase enzyme encoded by *FUT8* plays an important role in the glycosylation process and is responsible for adding fucose to glycoproteins and regulating the structure of cell-surface glycan chains. In cancer, FUT8 promotes the immune escape, proliferation, and metastasis of tumors by altering the pattern of tumor-cell surface glycosylation, making it a potential target for anti-tumor immunotherapy [61,62,63].

Stable prognostic stratification was achieved using the risk score model derived from seven glycolysis-related genes. The Kaplan–Meier survival assessment revealed a substantial divergence in OS across the elevated- and attenuated-risk cohorts. External validation across multiple independent datasets (e.g., BeatAML2.0, GSE37642, and GSE71014) further substantiated the stability and durability of the model in diverse cohorts of patients. The functional enrichment analysis confirmed that the AKT pathway was strongly activated in the elevated-risk cohort, while the PPI network suggested a central role for IL-6 within this group. IL-6, in turn, activates the AKT pathway, which promotes glycolysis, as has been shown in previous reports. These findings suggest that enhanced glycolysis activation in high-risk patients contributes to poor AML prognosis.

Besides serving as a central pathway in AML cell metabolism, the glycolytic pathway also modulates the immune microenvironment to impact prognosis. Analysis of immune infiltration revealed distinct immune cell profiles in the risk groups. The elevated-risk cohort showed high infiltration of monocytes and neutrophils, and the proportion of T cells (especially Th1 cells) was significantly decreased. A higher neutrophil-to-lymphocyte ratio (NLR) is commonly associated with worse prognosis, and a lower lymphocyte-to-monocyte ratio (LMR) is associated with increased neoplastic malignancy and adverse survival outcomes [64]. This immunosuppression in the microenvironment may lead to immune escape and promote leukemic cell proliferation, resulting in a poor prognosis for high-risk individuals. Glycolysis-mediated proinflammatory cytokine release may aggravate immunosuppression in the high-risk group, thereby facilitating immune evasion and disease progression, according to our results.

Drug sensitivity analysis showed that crizotinib and lapatinib are resistant in high-risk AML, whereas motesanib is highly sensitive in high-risk AML. Crizotinib is an ALK (anaplastic lymphoma kinase) mutation-targeting agent employed in the management of ALK-positive non-small cell pulmonary carcinoma (NSCLC) and various other neoplastic disorders. However, if MET inhibition increases dependence on oxidative phosphorylation and decreases dependence on glycolysis, cells may become more sensitive to crizotinib [65]. Lapatinib is a targeted therapy medication mainly utilized for HER2 (+) breast cancer and certain other cancers. Resistance to lapatinib is primarily driven by the activation of glycolysis, according to studies [66]. There is a limited known relationship between motesanib and glycolysis; however, as an angiogenesis inhibitor, motesanib is expected to also affect the availability of nutrients to tumor cells, thereby impacting tumor glycolysis. Together, these results imply that metabolic and immune heterogeneity has a strong impact on drug response in AML and suggest that more personalized treatment strategies are warranted. In particular, resistance to crizotinib seems to be intimately associated with metabolic adaptation, indicating that glycolytic pathway modulation may overcome treatment resistance. On the other hand, enhanced sensitivity to motesanib indicates that antiangiogenic and related pathways may have an important therapeutic role in patients at high risk of AML.

We integrated the GPM with the ELN classification to promote the prediction of AML prognosis. This integrated approach showed superior performance for high-risk patient identification, especially for patients with poor prognosis. These results highlight the crucial involvement of glycolysis in AML pathophysiology and offer a more accurate clinically applicable approach in risk stratification, enabling improved patient care and treatment delineation. The relationship between glycolysis and the prognosis of AML is revealed by this study, and the relationship between glucose metabolism and the mechanism of AML progression is further explored. Although the results of this study have potential applicability, there are areas for further improvement. Firstly, our prognostic model was developed using retrospective data from publicly available databases, which may limit its broader applicability. To enhance clinical utility, we plan to collect prospective clinical data in future studies for validation and further explore its potential for individualized therapy. In addition, while we have identified key roles for glycolysis in AML progression and immune regulation, the specific molecular mechanisms remain incompletely understood and warrant further experimental research. Our study primarily relied on transcriptomic data and lacked multi-omics integration, which may limit our full understanding of metabolic reprogramming in AML. Future studies incorporating multi-omics analysis will help improve the robustness of the model and provide deeper insights into the pathogenesis of AML. We also acknowledge that this study did not fully control for factors affecting OS, such as prior treatment regimens, comorbidities, and patient health status, which may influence survival outcomes. Future studies should include more comprehensive clinical data and minimize the impact of confounding factors using statistical methods like multivariate adjustment. Finally, one limitation of this study is the lack of experimental validation for the identified prognostic genes. In the future, we plan to conduct experimental studies using cell lines or patient samples to validate the roles of these genes in AML progression and assess their potential as clinical biomarkers.

## 5. Conclusions

This study demonstrates the pivotal role of the glycolytic pathway in disease prognosis and establishes a GPM for AML, offering a novel framework for personalized therapy. The integration of immune microenvironment features, drug sensitivity profiles, and the ELN classification enhances the risk stratification and individualized treatment approaches for AML, paving the way for the clinical application of glycolysis-based therapies. While this model offers significant information for the clinical management of AML and facilitates the formulation of customized therapy strategies, further validation through prospective studies is required.

## Data Availability

Public datasets were downloaded and analyzed in this study, including from the GEO database (accession numbers: GSE12662, GSE71014, GSE37642), TCGA-LAML cohorts, the GDSC database, the MSigDB compendium, and the BeatAML2.0 database.

## Supplementary Materials

The following supporting information can be downloaded at: www.mdpi.com/article/10.3390/biomedicines13040834/s1, Table S1. Potential therapeutic drugs targeting glycolysis-related genes in Acute Myeloid Leukemia. Figure S1. Kaplan-Meier survival curves based on quartile stratification of risk scores. AML patients were divided into four quartiles based on the glycolysis-related prognostic model (GPM) risk scores. The highest risk quartile exhibited significantly poorer overall survival compared to the lower quartiles (*p* < 0.01, log-rank test). Figure S2. Heatmap showing the association between risk groups and clinical parameters in AML patients. The heatmap illustrates the distribution of clinical features, including age, white blood cell count, and cytogenetic risk, among high-risk and low-risk groups in the TCGA cohort. Figure S3. Heatmap depicting frequent molecular and cytogenetic variations, as well as clinical indicators, in the 7-GPM high-risk group in the TCGA cohort. Specific mutations and cytogenetic abnormalities associated with poor prognosis are highlighted. Figure S4. Heatmap depicting frequent molecular and cytogenetic variations, as well as clinical indicators, in the 7-GPM low-risk group in the TCGA cohort. Key molecular markers associated with favorable prognosis are presented. Figure S5. Forest plot showing genetic abnormalities with differential incidences between high-risk and low-risk AML patient groups. The plot illustrates significantly enriched mutations and cytogenetic alterations in each risk category, emphasizing their potential prognostic significance (*p* < 0.05).
