# Supplementary material for: Glycolysis-Driven Prognostic Model for Acute Myeloid Leukemia: Insights into the Immune Landscape and Drug Sensitivity
Source: Biomedicines. 2025 Mar 31;13(4):834. doi: 10.3390/biomedicines13040834 (PMC12024913; doi:10.3390/biomedicines13040834)
Supplement: Supplementary file 1 [file biomedicines-13-00834-s001.zip › Supplementary information.pdf]

Supplementary Information

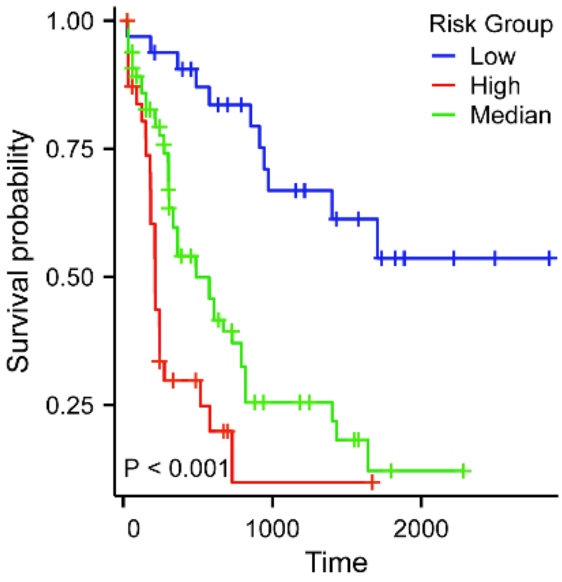

Figure S1. Kaplan-Meier survival curves based on quartile stratification of risk scores. AML patients were divided into four quartiles based on the glycolysis-related prognostic model (GPM) risk scores. The highest risk quartile exhibited significantly poorer overall survival compared to the lower quartiles (*p* < *0.01*, log-rank test).

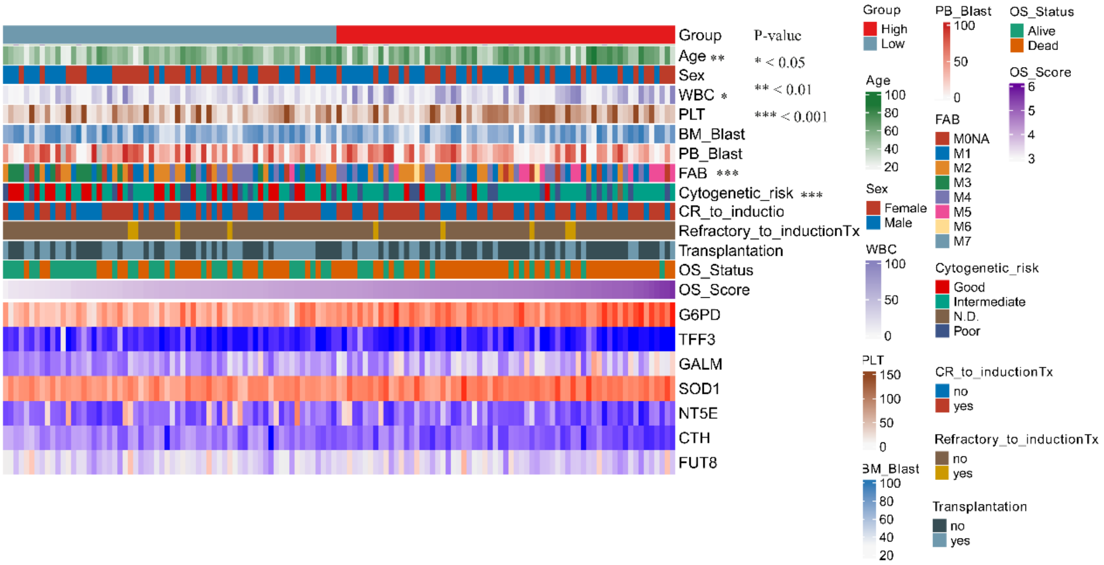

Figure S2. Heatmap showing the association between risk groups and clinical parameters in AML patients. The heatmap illustrates the distribution of clinical features, including age, white blood cell count, and cytogenetic risk, among high-risk and low-risk groups in the TCGA cohort.

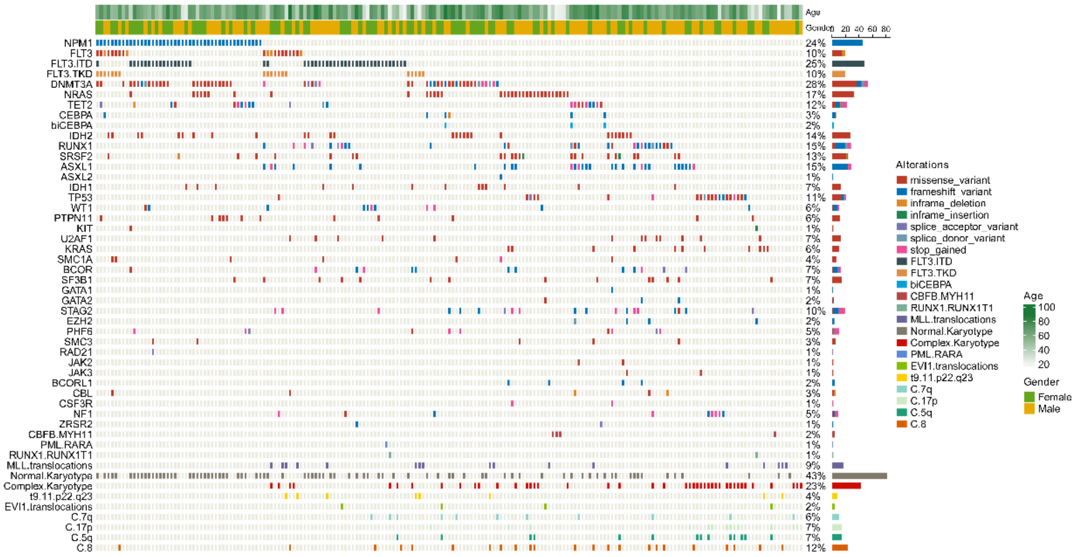

Figure S3. Heatmap depicting frequent molecular and cytogenetic variations, as well as clinical indicators, in the 7-GPM high-risk group in the TCGA cohort. Specific mutations and cytogenetic abnormalities associated with poor prognosis are highlighted.

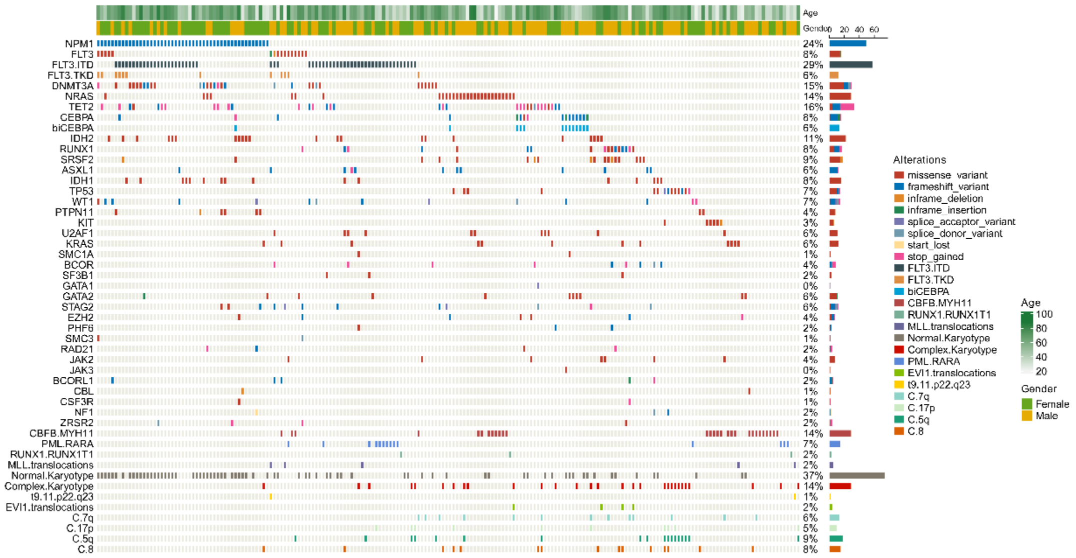

Figure S4. Heatmap depicting frequent molecular and cytogenetic variations, as well as clinical indicators, in the 7-GPM low-risk group in the TCGA cohort. Key molecular markers associated with favorable prognosis are presented.

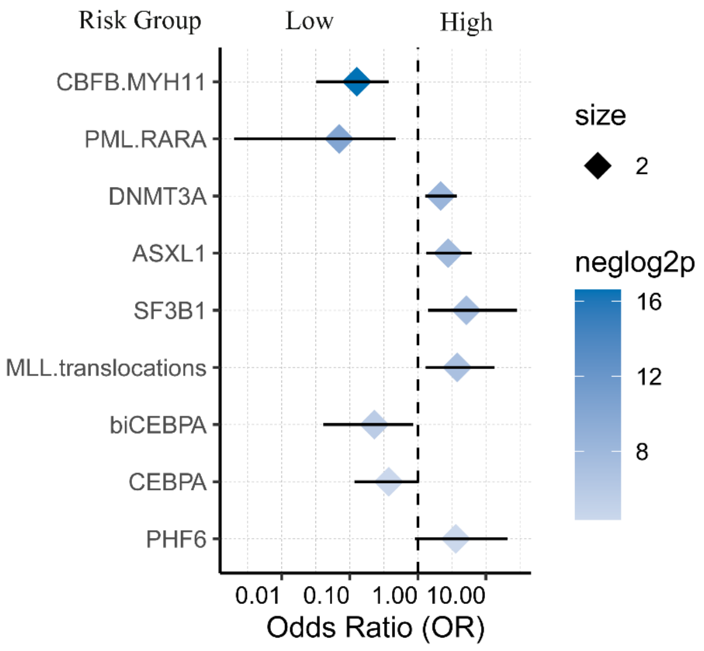

Figure S5. Forest plot showing genetic abnormalities with differential incidences between high-risk and low-risk AML patient groups. The plot illustrates significantly enriched mutations and cytogenetic alterations in each risk category, emphasizing their potential prognostic significance ($p < 0.05$).
